# Review: Bilirubin pKa studies; new models and theories indicate high pKa values in water, dimethylformamide and DMSO

**DOI:** 10.1186/1471-2091-11-15

**Published:** 2010-03-29

**Authors:** Pasupati Mukerjee, J Donald Ostrow

**Affiliations:** 1 School of Pharmacy, University of Wisconsin, 777 Highland Ave., Madison, WI, 53705-2222 USA; 2 GI/Hepatology Division, Department of Medicine, Box 356424, University of Washington School of Medicine, 1959 NE Pacific St., Seattle, WA 98195-6424, USA

## Abstract

**Background:**

Correct aqueous pKa values of unconjugated bilirubin (UCB), a poorly-soluble, unstable substance, are essential for understanding its functions. Our prior solvent partition studies, of unlabeled and [^14^C] UCB, indicated pKa values above 8.0. These high values were attributed to effects of internal H-bonding in UCB. Many earlier and subsequent studies have reported lower pKa values, some even below 5.0, which are often used to describe the behavior of UCB. We here review 18 published studies that assessed aqueous pKa values of UCB, critically evaluating their methodologies in relation to essential preconditions for valid pKa measurements (short-duration experiments with purified UCB below saturation and accounting for self-association of UCB).

**Results:**

These re-assessments identified major deficiencies that invalidate the results of all but our partition studies. New theoretical modeling of UCB titrations shows remarkable, unexpected effects of self-association, yielding falsely low pKa estimates, and provides some rationalization of the titration anomalies. The titration behavior reported for a soluble thioether conjugate of UCB at high aqueous concentrations is shown to be highly anomalous. Theoretical re-interpretations of data in DMSO and dimethylformamide show that those indirectly-derived aqueous pKa values are unacceptable, and indicate new, high average pKa values for UCB in non-aqueous media (>11 in DMSO and, probably, >10 in dimethylformamide).

**Conclusions:**

No reliable aqueous pKa values of UCB are available for comparison with our partition-derived results. A companion paper shows that only the high pKa values can explain the pH-dependence of UCB binding to phospholipids, cyclodextrins, and alkyl-glycoside and bile salt micelles.

## Background

Unconjugated bilirubin (UCB) in aqueous solution exists as an equilibrium among three species, the diacid (H_2_B), the monoanions (HB^-^) and the dianion (B^=^) [1]. Each species differs as to ionization states, properties and functions [1]. The fully protonated, uncharged, UCB diacid diffuses freely across lipid membranes [2,3]. The monoanion, with one ionized carboxylic group, is the main substrate for active cellular export of UCB by ABC-transporters [3]. The dianion, with two ionized -COO^- ^groups, is reported to be bound preferentially with high affinity to serum albumin [4,5], apolipoprotein-D [6], and ligandin and other GSH-transferases [7], as well as to bile salts [8].

Since the relative proportion of the three species depends on the pH of the solution and the pKa values of UCB [1], the true pKa values of UCB are of great physiological and basic relevance. There are, however, tremendous variations among the reported pKa values for bilirubin in aqueous solutions, as determined by a wide variety of methods (table eight in Boiadjiev *et al. *[9]). Most studies in the literature suggested pKa values below 7.0 and even below 5.0 [9], whereas our solvent partition studies [10,11] indicated that the two pKa values were much higher, 8.12 and 8.44. The variations in reported pKa estimates are due in large part to the methodological difficulties of studying UCB at concentrations below its low aqueous solubility limit (< 0.1 *μ*M at pH ≤ 7.8 [12]) and the ready degradation of the pigment to more polar derivatives with much higher solubility and different ionization properties [13-15].

The choice of pKa values also affects estimates of the aqueous solubility of UCB [12]. If the assumed pKa values of 4.4 and 5.0 [16] are used to represent low pKa's, the ratio of diacid/dianion at pH 7.4 would change from 0.58 (high pKa's) to only 4 × 10^-6 ^(low pKa's), and the solubility of UCB diacid would change from the experimental value of 5 × 10^-8 ^M [10] to less than 10^-14 ^M [12]. Such differences are clearly of great significance in understanding the interactions of UCB with proteins and cell membranes [1] and its protective and toxic effects on cells [3].

We have critically re-examined many conflicting accounts of the pKa data and have assessed the reliability of the methods used, in relationship to minimal criteria (detailed below) as well as other considerations. In this paper, we examine studies that reported pKa values of UCB in simple aqueous systems, pure organic solvents, or mixtures of organic and aqueous solvents (Additional File 1, Table S1). The effects of varied pH on the binding of UCB to phospholipids, dodecylmaltoside micelles, cyclodextrins, and bile salt micelles are considered in a companion paper [17]. Extensive reinterpretations of the published data, employing some new models, reveal that these data are compatible with the high aqueous pKa values for UCB and incompatible with low pKa values.

## Methods

### Assessment Criteria for Validity of Studies

By definition, pKa values can only be determined if the free, aqueous phase UCB concentrations are below saturation and monomeric; any UCB aggregates formed at higher concentrations must be measured and accounted for. The propensity of UCB to deteriorate requires that: the pigment is purified just before experimental use [15], it is dissolved and studied under conditions that minimize exposure to high pH, light and oxygen, and that the measurements are made over a brief time span. These minimal criteria must be met if pKa determinations are to be valid.

The full criteria used in assessing such studies are: 1) The UCB has been purified and its purity documented by spectrometric and chromatographic methods [13]; 2) There should be no significant degradation of UCB, to polar derivatives with low pKa values, during its dissolution or storage in concentrated stock solutions, or during the incubation and analyses [13]; 3) Measurements are made after equilibrium is achieved, but the equilibrium should be attained rapidly to minimize degradation of UCB; 4) The pH range examined should ideally be wide enough to encompass all suggested pKa values of UCB (pH 4 to 10), and include sufficient data points for mathematical modeling; 5) Since thermodynamic theory defines pKa as an equilibrium among dissolved, monomeric species, the study should be done at unbound UCB concentrations below to minimally above aqueous saturation, must not be confounded by the presence of insoluble aggregates of H_2_B, and must include a measurement and accounting of any soluble multimers (such as B^= ^dimers) [10]. To assess the likelihood of the involvement of colloidal and coarser particles, we have calculated the supersaturation ratio, R, which is the free UCB concentration divided by the estimated solubility of UCB at a given pH (e.g. 55 nM at pH 4) [10,12]. R is, in effect, a measure of the driving force for nucleation and growth of particles.

### Selection of Publications for Further Analysis

To find papers for possible review, we electronically searched PubMed (1967-date) and ISI and Chemical Abstracts databases back to 1950, using the keywords "bilirubin, hydrogen-ion concentration, pH, pKa", as well as the reference lists in papers thus discovered. To locate papers published earlier than 1968, we manually searched T.K. With's two comprehensive compendia of studies related to bilirubin [18,19]. Papers were eliminated that dealt only with: bile pigments other than biladienes, bilirubin ester conjugates, or effects of pH on binding of UCB to other molecules. We then selected all papers that derived pKa values from their data and met the majority of the criteria of validity summarized above, plus others that did not, but are frequently quoted (see table eight in Boiadjiev *et al. *[9]) and therefore required comment.

Additional File 1, Table S1 lists and summarizes the 18 studies and indicates the experimental limitations of each study. Of these 18 studies: four utilized spectrometric titration of highly supersaturated solutions of UCB [20-23]; two used potentiometric titration of supersaturated solutions of UCB [16,24] (the former [16] also assumed low pKa values to model the data, the other included Triton-X100 detergent in the system); one performed potentiometric titration of a water-soluble, thioether conjugate of polyethylene glycol monomethyl ether with UCB (MPEG-S-BR) [9]; two studied UCB crystal dissolution vs. pH [22,25] (the problems of such inherently supersaturated systems are discussed in Hahm, *et al. *[10]); two derived aqueous pKa values from studies of UCB in dimethylformamide (DMF) [26] or DMSO [27]; four studied [^13^C]-labeled mesobilirubin-XIIIα in mixed solvent systems of DMSO and water [28-31]; and three measured solvent partition of UCB between an organic solvent phase and water over a range pH values [10,11,32] (the earliest [32] used systems very supersaturated with UCB at all pH values and assumed low pKa values to model the data).

## Results and Discussion

### Studies of UCB in Simple Aqueous Systems

#### Spectroscopic Titrations

Spectral changes have been reported when aqueous solutions of bilirubin at high pH are acidified rapidly [33,34] or gradually [35]. Formation of colloidal aggregates and coarser particles from supersaturated solutions can produce spectral changes similar to those produced by acidification and may vary with aging [33,34]. Such variability is to be expected because of kinetic control of processes such as homogeneous or heterogeneous nucleation, and growth and flocculation of colloidal particles [36]

At 0.3 *μ*M, with by far the lowest R value of 5.5 at pH 7.0, Hansen *et al. *[27] reported that there was no immediate spectral change as pH was varied from 9 to 7, but light scattering increased over 30 minutes at pH 7. At higher UCB concentrations, however, they observed spectral changes, accompanied by an increase in light scattering, as pH fell from 8 and 7. Lee and Gartner [34] worked at the modestly higher R of 15.5 at pH 7.0, in the presence of the antioxidants, ascorbate and EDTA. As pH increased, they observed a relatively steep sigmoidal increase in A_440 _of UCB in phosphate buffer, with a titration midpoint at pH ~7.5. These investigators did not derive any pKa values from the titration midpoint. Using a much more saturated system (R~275), Gray *et al. *[20] reported that the absorbance of UCB at the band maximum decreased as pH fell from 8 to 6, and they estimated the average pKa to be about 7.1. These variable spectral changes are consistent with formation of colloidal or coarser particles [27,33,34].

At a concentration of 1.4 *μ*M (R = 25), Moroi *et al. *[22] estimated a pKa_2 _value of 7.3-7.6 and a pKa_1 _of 6.1-6.5. Russell *et al. *studied changes in the vibrational (resonance Raman) and electronic (UV-Visible) spectra of UCB [23]. On lowering the pH from 10.0 to 7.4 in aqueous solutions, they found an inflection point at pH 8.3, and deduced a high pKa_2 _value of 8.3. They reported also that "Under these conditions the titration was reversible and no precipitation was observed. This was confirmed by comparing spectra obtained before and after filtration." However, while stable supersaturation is possible, its relief leading to phase separation may not lead to particles large enough to be removed by ordinary filtration [12]. At lower pH values, the authors found that precipitation occurred. They estimated an approximate value of about 6 for pKa_1_.

Kolosov and Shapovalenko [21], in a paper lacking many experimental details, reported that absorbance at 430 nm decreased by about 31% as pH decreased from 8.5 to 6.5 and by about 15% as pH decreased from 5.5 to 4.5. Three sets of pKa values were invoked, 5.2 for pKa_1_, 5.9 for pKa_2 _and 7.3 for the average of pKa_3 _and pKa_4_. These latter pKa values would suggest that bilirubin exists primarily as a tetra-anion at pH 8, an unlikely possibility [27]. Recent authors [37] noted these low pKa_1 _and pKa_2 _values, but did not mention the pKa_3 _and pKa_4 _values obtained from the same titration.

In summary, spectroscopic titrations in water at pH 8 or below have yielded a wide range of estimated pKa values (Additional File 1, Table S1), but none have been carried out both with purified UCB and in undersaturated solutions. The spectral changes observed were likely due mainly to the formation of colloids and coarser aggregates, and are therefore not relevant to, or valid for, estimation of pKa values in an undersaturated solution of UCB monomers.

#### Potentiometric Titrations

In an early study, Overbeek *et al. *[16] dissolved a UCB suspension by adding NaOH, then performed a potentiometric titration with HCl, followed by a backtitration with NaOH. In order to interpret their titrations, they assumed pKa_1 _and pKa_2 _values of 4.4 and 5.0. Lucassen [38] encountered serious difficulties in trying to reproduce these experiments of Overbeek *et al. *Krasner and Yaffe [24] reported a pKa value of 7.55 from potentiometric titrations with both HCl and NaOH. Hansen *et al. *[27] obtained very similar titration curves. Because of massive supersaturation and extensive precipitation, however, no reliable pKa values can be derived from these titrations, as emphasized by Hansen *et al. *[27], Lee *et al. *[26], and Carey [35].

#### Solubility vs. pH

Ostrow *et al. *[25] studied the dissolution of UCB crystals in buffers. They found that stable UCB concentrations were achieved only after 48 hours and varied little over the pH range 4 to 6. Modeling the data yielded pKa_1 _and pKa_2 _of 6.8 and 9.3 with a high average pKa of 8.1. They pointed out some sources of uncertainties, particularly in the estimate of the low solubility of uncharged bilirubin diacid. This was also noted as a major problem by Moroi *et al. *[22], who thought that their estimate of pKa_2 _from solubility data, 7.6, was more reliable than their estimate of pKa_1_, 6.0. Subsequently, the numerous difficulties of the solubility method, arising out of crystal imperfections, fine particle solubility effects, difficulty of equilibration and Ostwald ripening, have been discussed theoretically and documented experimentally [10]. These problems render doubtful the validity of such estimates of pKa values.

### Bilirubin in Systems Containing Organic Solvents

To circumvent the problems created by the low aqueous solubility of UCB, several investigators have attempted to indirectly estimate pKa values in water from titrations in organic solvents in which the solubility of UCB is high, dimethylformamide (DMF) [26] and DMSO [27]. Neither of these studies, however, involved any direct measurements in water. As mentioned below, DMSO-water mixtures have also been used [29,37].

#### Titrations in DMF

A seldom-used method, of estimating pKa values in water from measurements of half-neutralization potentials (HNPs) in an organic solvent, was applied by Lee *et al. *[26] to UCB in DMF. The difference in HNP_1_, corresponding roughly to pKa_1 _in DMF, from the HNP of benzoic acid, ΔHNP_1_, was found to be linearly related to pKa_1 _values in water of four reference dibasic acids, succinic, glutaric, adipic, and azelaic. Using this reference curve, the measured ΔHNP_1 _for UCB in DMF was used to estimate its pKa_1 _in water. Similarly, the difference between HNP_2 _(corresponding to pKa_2_) and HNP_1 _for UCB in DMF was related to the difference of pKa_2_-pKa_1 _for the reference acids, leading to the estimate of pKa_2_-pKa_1 _of UCB in water from the measured HNP_2_-HNP_1 _measured in DMF. This method yielded low values of 4.3 for pKa_1 _and 5.3 for pKa_2 _for UCB in water [26].

This indirect approach is based on some rough correlations reported in 1958 by Streuli and Miron [39], who measured HNP_1 _values for 44 carboxylic acids in pyridine. They reported three very different groups of correlations of HNP_1 _with aqueous pKa values and numerous deviations from each of them, particularly for acids with intramolecular hydrogen bonding. Ortho-hydroxybenzoic acid, for example, deviated from the correlation line of other ortho-substituted benzoic acids by 1.5 pKa units. From the correlation line between HNP_1 _and six dicarboxylic acids similar to the four used by Lee *et al. *[26], maleic and phthalic acids differed by 1.8 and 0.9 units respectively. Similarly, maleic acid showed a deviation of 2.9 units from the correlation of HNP_2_-HNP_1 _with pKa_2_-pKa_1 _of the reference dibasic acids. Maleic acid, with such high discrepancies, has only one intramolecular H-bond in the monoanion, whereas uncharged UCB diacid has six such bonds. We believe that deviations caused by the complex intramolecular hydrogen bonding of UCB cannot be easily evaluated or ignored, and that this highly indirect ΔHNP method, demonstrably unreliable for estimating pKa values of simple acids in water, is unlikely to be reliable for a molecule as complex as UCB. In DMF, ΔHNP_1 _for UCB is closest to that of succinic acid, for which Kolthoff *et al. *[40] had directly measured very high pKa_1 _and pKa_2 _values in DMF of 10.05 and 17.21, respectively. Therefore, pKa values of UCB in DMF are probably higher than 10.05 and thus considerably higher than our partition-derived values of 8.12 and 8.44 for UCB in water [10]. This is in keeping with the observation that pKa values of carboxylic acids are higher in DMF than in water [41].

#### Titrations in DMSO

Based on extrapolation to 0% DMSO from [^13^C]-NMR measurements of -[^13^C]OOH group ionizations of mesobilirubin in varied mixtures of DMSO and water, the two pKa values of mesobilirubin in water have been reported to be 4.2 and 4.9 [9,29,30,37]. Due to the demonstrated problems of insolubility [12], large errors in pH measurements in the mixed solvents [31,42], and the long, even overnight duration of the [^13^C]-NMR analyses [29], these studies are not interpretable.

Hansen *et al. *[27] estimated an average pKa value of 4.4 for UCB in aqueous solutions from comparison of titrations of UCB and m-hydroxybenzoic acid performed in DMSO. Using the Born equation [27], assuming that all ions are spherical, and arbitrarily setting the radii of bilirubin IXα, m-hydroxybenzoic acid, and the solvated proton to be 7, 2, and 2Å, respectively, they calculated the pKa of m-hydroxybenzoic acid in DMSO to be 5.1 from its known aqueous pKa value of 4.0 [27]. Since "Potentiometric cotitration of bilirubin and m-hydroxybenzoic acid revealed that the carboxylic acid pKa's of bilirubin and m-hydroxybenzoic acid are identical in dimethyl sulfoxide within experimental error ...", the pKa of UCB in DMSO was estimated to be 5.1 also. The Born equation was then used to calculate the average pKa of UCB in water to be 4.4. It is well-known that the acid-base character of solvents and several other factors are of much greater importance in determining pKa values than the purely electrostatic interactions of spherical ions covered by the Born equation [41,43]. Indeed, the directly measured pKa value of m-hydroxybenzoic acid in DMSO is 11.1, [44], which is 6 units higher than the value of 5.1 calculated by Hansen *et al. *some years later [27]. Benzoic acid and *m*-methyl benzoic acid have a similar pKa of 11.0 in DMSO [44]. The indisputable experimental identity of pKa values of m-hydroxybenzoic acid and UCB in DMSO thus leads to a firm conclusion that the average pKa of UCB in DMSO is about 11.1, which is notably about 3 units higher than the average pKa of 8.3 in water, derived from our partition studies [10]. This is consistent with the finding that carboxylic acids of many kinds have higher pKa values in DMSO than in water [41]. Therefore, the low estimate of 5.1 for the average pKa of UCB in DMSO, and the estimated average pKa of UCB in water of 4.4, calculated therefrom by use of the Born equation and arbitrarily chosen radii of the ions, are both unacceptable. We apologize for having accepted this erroneous value in the past [25], because we paid insufficient attention to the earlier work of Kolthoff *et al. *[44].

There are large but variable solvent effects associated with molecules in which H-bonds donated by carboxyl groups are broken on ionization (i.e. the second dissociation of dicarboxylic malonic or maleic acids) and with molecules such as salicylic acid, in which neighboring non-carboxylic groups donate H-bonds to a carboxyl group [41]. Uncharged UCB diacid has a unique, complex combination of H-bonds donated by and accepted by its carboxyl groups [1], and no simple molecular analogue is available. A quantitative interpretation of the relative pKa values in water and DMSO is, therefore, not feasible currently.

#### Solvent Partition

Irollo *et al. *[32] studied the variation with pH of the partition of unpurified UCB from Tris-buffered water into varied mixtures of unpurified methyl-isobutyl ketone and n-heptane. As summarized in Additional file 1, Table S1, this study failed to meet many of the essential criteria of validity. Most importantly, the aqueous phase was supersaturated with UCB throughout the narrow pH range (7.6-9.0) studied (R was above 50 at pH 7.6), often with formation of visible precipitates. No pKa value can be derived from this data; indeed the authors modeled the data by assuming pKa values of 4.3 and 5.3 [26].

Our complimentary studies of solvent partition (from chlorofom into water) with unlabeled [10] and [^14^C]-UCB [11] are the only studies to have been performed with highly purified UCB at concentrations uniformly below its solubility limits at all pH values. The concordance of results between the two studies refute criticisms [37] that the diazo-based assays of unlabeled UCB in the first study were inaccurate, insensitive, or non-specific for UCB. In both studies, degradation of UCB was minimized by performing the partitions rapidly, in the dark, under an argon atmosphere. The partitions utilized highly purified water, with chloroform that had been properly washed and stored to eliminate oxidative species that could rapidly degrade UCB [45,46]. Achievement of equilibrium was documented by reverse partitions from water into chloroform [10], and by performance of serial partitions to a constant partition ratio [11]. The constancy of the partition ratios at a given pH over a wide range of concentrations excluded significant aggregation of UCB diacid in the chloroform phase, and the model used took into account the self-association of the UCB dianion in the aqueous phase at high pH values [10]. Finally, the partitions were done over a pH range from 4 to 10, encompassing the entire range of proposed pKa values from the literature. These studies thus fulfilled all the key criteria, outlined above, for a valid evaluation of the aqueous pKa values of UCB.

### Water-Soluble Conjugates of Bilirubin and Dicarboxylic Fatty Acids with Polyethylene Glycol Monomethyl Ether (MPEG)

Boiadjiev *et al. *[9] performed NaOH titrations on a water-soluble conjugate of bilirubin (MPEG-S-BR), prepared by linking the *exo*-vinyl group of UCB through a thioether bridge to MPEG (average Mol. Wt. = 1900, a 42-mer). NMR data of UCB and MPEG-S-BR dissolved in (CD_3_)_2_SO and CDCl_3_, suggested that "the presence of the pendant polymer does not disrupt the stabilizing network of six intramolecular hydrogen bonds" in UCB. The titration with NaOH of MPEG-S-BR at a high nominal concentration of 8 mM in water showed a pH value of 6.42 at the midpoint of the titration. This apparent average pKa value is midway between the average of the low pKa values, 4.55 [9] and that of the high pKa values, 8.28 [10]. UCB is known to be highly aggregated at such a high concentration [9,10,47]. Carey and Koretsky concluded that, at pH 10, 270 *μ*M UCB is present mostly as multimers, including mixed aggregates of B^= ^and HB^- ^[47]. Boiadjiev *et al. *[9] were unable to detect [^13^C]- or [^1^H]-NMR signals from the bilirubin moiety of MPEG-S-BR dissolved in D_2_O. This was attributed to self-association of MPEG-S-BR into large micellar aggregates [9]. Clearly, the apparent pKa values from the midpoint of the titration curve cannot represent the pKa of monomeric UCB. There is also no direct evidence that monomeric UCB and monomeric MPEG-S-BR have identical intramolecular H-bonds in water.

Boiadjiev *et al. *[9] argued that the apparent average pKa of 6.42 for MPEG-S-BR provides evidence for the low pKa values of UCB by comparing the titration of MPEG-S-BR to the titrations of some water-soluble MPEG monoesters of dicarboxylic fatty acids, MPEG-FAs = MPEG-OCO-(CH_2_)_n_-COOH (n = 2,6,11,14 or 18). The long-chain MPEG-FAs, which are expected to show extensive micelle-type aggregation, were proposed as reference models for MPEG-S-BR. We provide some arguments and evidence indicating that the MPEG-FAs are poor models for MPEG-S-BR.

In aqueous solutions, relatively hydrophobic, amphipathic molecules of different structures follow very different patterns of self-association [48-50]. Classical amphipaths, such as sodium dodecyl sulfate or sodium laurate, have flexible aliphatic chains, which can form the liquid cores of micelle-type aggregates, the polar groups remaining at the surface [51]. The self-association is highly co-operative [48,51], resulting in the phenomenon of a critical micellization concentration (c.m.c.) [52]. In sharp contrast, rigid, planar, aromatic molecules such as methylene blue, which cannot form liquid-like cores in aggregates, show extensive self-association of the stacking type, with little co-operativity and, therefore, no c.m.c. [48,53,54]. Flexible-chain detergent-type molecules are thus very poor models for rigid, planar molecules, and are not even good models for bile salts, which have alicyclic, rigid structures [49,50]. UCB, depending upon the relative dispositions of the two dipyrrolic halves of the molecule, can have many conformations [55]. Different conformations, and ranges of conformations and shapes, may be expected for the H_2_B, HB^- ^and B^= ^species, and their corresponding MPEG-S-derivatives. The longer chain MPEG-FAs containing flexible aliphatic chains should resemble detergent-like amphipaths. MPEG-S-BR is unlikely to do so any more than methylene blue [48,53].

The unusual characteristics of the H_2_B, HB^- ^or B^= ^species are displayed by their uptake into the hydrophobic environment of the anionic bile salt micelles in 50 mM sodium taurocholate (NaTC) [8,56]. Charge effects render interactions of anions (A^-^) with such aggregates less favorable than the ordinary acid (HA), as is usually observed (e.g. fatty acids with cholate [57]). The micelle-water distribution ratios in 50 mM NaTC of H_2_B, HB^- ^or B^= ^(D_0_, D_1 _and D_2_, respectively), have increasing values, however, with increasing ionization (1.4 for H_2_B, 13 for HB^- ^and 730 for B^=^) [8]. The increasing charge repulsions expected in the NaTC micelles must thus be more than compensated by an increasing expression of hydrophobicity (H_2_B < HB^- ^< B^=^). If the acid dissociation constants of H_2_B and HB^- ^are K'_1 _and K'_2 _in the micellized state and K_1 _and K_2 _in aqueous solution, it is easily shown from the schemes presented [56-58] that K'_1_/K_1 _= D_1_/D_0 _and K'_2_/K_2 _= D_2_/D_1_. Thus, for UCB, D_1 _> D_0_, K'_1 _> K_1 _and pK'_1 _< pK_1_; similarly, pK'_2 _< pK_2_. The average pKa values of UCB in 50 mM NaTC aggregates are in the range of 6 to 7 [8,17,57]. These indicate that the pKa values of UCB in water, must be higher and not lower, as has been claimed [57]. This is more fully discussed in our companion paper [17]. The extraordinary increase in hydrophobic interactions with increasing ionization of UCB is also in accord with the extensive self-association displayed by UCB at high pH, where the B^= ^dianion predominates [9,10,47]. Some remarkable differences we have noted in the titration curves reported for MPEG-S-BR and the long-chain MPEG-FA derivatives [9] can be rationalized on this basis, as shown below.

The long-chain MPEG-FAs are expected to produce micelle-like aggregates, for which a general equation dealing with charge effects on pKa_(s) _(pKa of an acid group at the micellar interface) [59] can be adapted:(1)

Here, the pKa_(s) _at 25°C, is determined by the absolute value of the electrostatic potential |ψ| at the micellar interface, expressed in millivolts, and pK_i(s)_, the intrinsic pK_(s) _value when charge effects are absent (|ψ| = 0). An approximate estimate of |ψ| at 25°C when counterions are monovalent (e.g. Na^+^), can be obtained from the Gouy-Chapman theory of electrical double layers [60] by using Equation 2.(2)

where A is the area at the interface in sq. Å/charge and c is the molar concentration of the counterions. Equations 1 and 2 show that, with increasing neutralization with NaOH, as progressive ionization of the -COOH groups increases the charge density (surface potential) at the micellar-aqueous interface [59], the value of A decreases and the values of |ψ| (Equation 2) and pKa_(s) _(Equation 1) should increase.

Only rather qualitative applications of Equations 1 and 2 are possible here. The titration curves of the MPEG-FAs [9], show some anomalous features. The expected pH values of 11.0-11.1, calculated from the excess NaOH added beyond the identified neutralization points, are 1.4-1.6 units higher than the measured pH values, read from the graphs. This suggests incomplete neutralization. In addition, inappropriately high molecular weights of the MPEG-FA derivatives are calculated from the NaOH equivalents at the assumed titration end-points and the initial weighed amounts of each derivative. Thus, from the mol. wt. of MPEG used, about 1900, the mol. wts. of the esters should be below 2300. The values calculated from the titration equivalents are much higher for the suberic, brassylic, thapsic and eicosanedioic derivatives (4633, 6866, 3969 and 5270 respectively). This indicates also ill-defined preparations and/or premature assignment of titration end-points. The calculations below are thus of qualitative significance only.

Using the published titration curves for MPEG-FA in Figure three of Boiadjiev, *et al. *[9]) the overall apparent pKa values of the MPEG-FA at differing degrees of neutralization can be calculated from the pH values read off the graph and the estimated [A^-^/]/[HA] ratios. These apparent pKa values show the trends, expected from Equations 1 and 2, to increase with progressive neutralization and charge build-up in the micelles. For the brassylic, thapsic and eicosanedioic acid derivatives respectively, the apparent pKa values are 5.0, 5.2 and 5.5 at 5% neutralization, 5.26, 5.94 and 6.60 at 50% neutralization, and 6.2, 6.5 and 7.4 at 95% neutralization. In the absence of detailed information about the free monomer concentrations, aggregate structures, and A values, quantitative calculations using Equation 2 are not possible. If we make the simplifying assumptions of complete aggregation and 60 sq. Å/charge at full neutralization, the value appropriate for dodecyl sulfate micelles [61], and assuming pK_i(s) _of 4.6, the pKa value of MPEG-O-succinate, we calculate pK_(s) _values of 7.1, 8.2 and 8.6 at 5%, 50% and 95% neutralization for MPEG-O-eicosanedioate. This agrees in trend and order of magnitude with the corresponding pK_(s) _values of 5.5, 6.6 and 7.4 estimated from the experimental data. The titration curves of the flexible chain MPEG-S-FA systems thus appear to be well within the bounds expected from well-established theories of charge effects in interfacial systems. This, however, is not the case for the MPEG-S-BR titration curves, as shown below.

For a dibasic acid such as UCB, the pH and Na^+ ^concentrations at any point on the titration curve can be used to calculate the apparent average K_1 _and K_2 _values, using two equations:(3)(4)

The extremely minor contributions of [H^+^] and [OH^-^] in the charge balance Equation 4 can generally be ignored. If we make the reasonable assumption that K_1 _= 4K_2 _[41,43], simultaneous Equations 3 and 4 can be readily solved for either K_1 _or K_2_. From the estimated pH and [Na^+^] at 5% and 95% neutralization of MPEG-S-BR with NaOH [9], we calculate pK_1 _and pK_2 _values of 6.1 and 6.7 at 5% neutralization and very similar values of 5.9 and 6.5 at 95% neutralization, the estimation uncertainties being about 0.2. From 5% to 95% neutralization, the charge per molecule increases by 0.9 for the monobasic MPEG-O-eicosanedioate, and the apparent pKa increases by 1.9 units (see above). In the case of MPEG-S-BR, a dibasic acid, the charge increases by twice as much, 1.8 units per molecule, and yet there is almost no change in pKa values. The charge effects on pKa values of MPEG-S-BR are clearly inconsistent with the micelle model.

The reverse titration of the salt of MPEG-S-BR with HCl reveals another remarkable inconsistency with the NaOH titration. The pH value at 5% titration (i.e. 95% neutralization) is about 10.1, leading to a pK_2 _estimate of about 9.1, a very high value. In this titration, a gentle reduction in pH with the initial addition of HCl is followed by a precipitous decrease in pH with an inflection point, well into the titration, but before the midpoint. No such behavior is reported with the titration of MPEG-S-BR with NaOH.

Acid-base equivalences calculated from the titration data for MPEG-S-BR are also remarkable. The amount of MPEG-S-BR used in the NaOH titration, using the mol. wt. of 2520 given for MPEG-S-BR [9], corresponds to 0.400 mEq. Only 0.305 mEq of NaOH (24% less) was added at the assumed titration end-point. Further addition of about 0.64 mEq of NaOH raised the pH to only 10.5, compared to the pH of 11.0 expected from addition of the base to a fully neutralized solution at the assumed endpoint. This indicates that there was incomplete neutralization at the assumed end-point and/or significant problems with homogeneity and purity. The back-titration of the salt of MPEG-S-BR with HCl used 0.345 mEq, which is 13% higher than the 0.305 mEq of NaOH used for the titration of the acid, and 14% lower than the calculated initial amount (0.400 mEq). These discrepancies are much too large for simple acid-base titration experiments. These serious inconsistencies in the titration data for MPEG-S-BR and their dissimilarity with such data for MPEG-O-eicosanedioate, and similar amphipaths with flexible aliphatic chains, render questionable an assumption that these hydrophobic solutes behave similarly when aggregated. Thus, any conclusion about the pKa values of monomeric UCB derived from the pH of the midpoint of the titration of MPEG-S-BR with NaOH is also questionable.

In order to shed some light on the apparently anomalous titration behavior of MPEG-S-BR, we examine now some possible consequences of what is known qualitatively about the self-association of UCB to the titration behavior of MPEG-S-BR, making the simplifying assumption that it behaves like UCB. For four different models of self-aggregation of the UCB dianion, we calculated the pH values expected from titration of UCB with NaOH at concentrations similar to those used for MPEG-S-BR by Boiadjiev *et al. *[9]. pH values were calculated using the assumptions and equations presented in the Appendix, and are plotted against F, the ratio of the equivalents of added NaOH to the equivalents of UCB. The equivalence point corresponds to F = 1. We have used our previous estimates for UCB of pKa_1 _= 8.1, pKa_2 _= 8.4, and K_D _= 2.6 × 10^5 ^M^-1^, the formation constant for the dimer of B^= ^[10]. Although extensive multimerization of UCB has also been indicated [9,47], the multimers have not been characterized. We have, therefore, assumed the formation of only some multimers, strictly for qualitative modeling.

**Figure 1:**
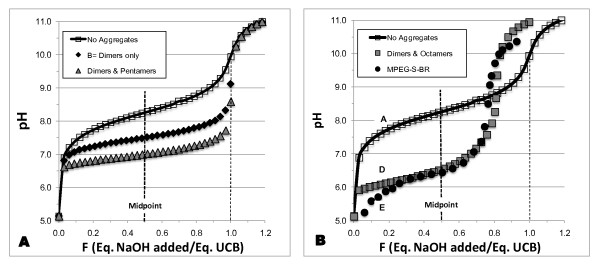
**Influence of aggregates on the titration of UCB with NaOH**. Four models of changes in pH expected during titration of 25 ml of 7 mM UCB with 10.6 mM NaOH, compared with the experimental titration curve of MPEG-S-BR [9]. pH values, calculated according Equations 5 and 6 in the Appendix, are plotted against F, the ratio of the equivalents of added NaOH to the equivalents of MPEG-S-BR. Full neutralization corresponds to F = 1 (light dashed line) and the titration mid-point is at F = 0.5 (heavy dashed line). The models apply our previously estimated constants for UCB [10] of pK_1 _= 8.1, pK_2 _= 8.4, and K_D _= 2.6 × 10^5 ^M^-1^, the formation constant of (B^=^)_2_, the dimer of the UCB dianion. The models considered below ignore the even higher multimers of B^= ^and higher mixed aggregates of HB^- ^and B^= ^that would be expected in the real system [9,47]. See Appendix for details. **A**. Curve A (open squares) assumes there is no self-association of any UCB species. Curve B (black diamonds) assumes the only aggregate is the dianion dimer, (B^=^)_2_. Curve C (gray triangles) assumes dimers and pentamers, the added pentamer species, (B^=^)_5_, having a formation constant K_5_, given by log K_5 _= 22.66 for the equilibrium 5 B^= ^↔ (B^=^)_5_. **B**. Curve D (gray squares) incorporates (B^=^)_2_, and three octamers, (H^+^)_3_(B^=^)_8_, (H^+^)_4_(B^=^)_8_, and (H^+^)_5_(B^=^)_8_, which are mixed adducts of HB^- ^and B^= ^with formation constants given by log K = 40, 39 and 38, respectively. Curve E (black dots), the experimental potentiometric titration curve of MPEG-S-BR from Figure 2a of Boiadjiev *et al. *[9] (see text), is approximated by Curve D, but not by Curve A. The pH at the titration mid-point for each curve (A, 8.26; B, 7.62; C, 6.99; D, 6.54) decreases as the size of the UCB aggregates increases, and is lowest for MPEG-S-BR (E, 6.42).

Figure 1 A&B shows the calculated titration curves. If there is no self-association (curve A), the pH at the midpoint, 8.25, is expected when pKa_1 _and pKa_2 _are 8.1 and 8.4. When the only aggregate is (B^=^)_2_, the titration occurs at lower pH values and the midpoint pH is lowered quite significantly to 7.52 (curve B). For curve C, we use a dimer-pentamer model, the added pentamer species, (B^=^)_5_, having a formation constant K_5_, given by log K_5 _= 21.66 for the equilibrium 5 B^= ^<---> (B^=^)_5_. The midpoint pH is depressed further, to 6.99, approaching the value of 6.42 estimated for the titration of MPEG-S-BR with NaOH [9]. As noted above, in the real system, higher multimers of B^= ^and mixed aggregates of HB^- ^and B^= ^would be expected. In Figure 1B, curve D, we show a model containing (B^=^)_2_, and mixed aggregation of HB^- ^and B^= ^to produce the octamers, (H^+^)_3_(B^=^)_8_, (H^+^)_4_(B^=^)_8 _and (H^+^)_5_(B^=^)_8_. As explained in the Appendix, we assume the formation constants of these aggregates to be given by log K = 40, 39 and 38 respectively. When the three octameric species are added to the dimer, the calculated titration curve D shows some remarkable features that closely mimic the very peculiar characteristics of the published titration curve (E) for MPEG-S-BR [9]. For this curve, we assumed MPEG-S-BR to be pure and calculated F values from the mEq of NaOH added to 0.400 mEq of MPEG-S-BR [9]. The steep increase in pH with added NaOH around F = 0.80, 20% below nominal neutralization is extraordinary and can easily lead to a premature end-point assignment. It simulates the steep increase observed for MPEG-S-BR which leads to an assigned titration end-point about 24% below the nominal neutralization point. The pH changes in gentle fashion at around F = 1. The mid-point pH value is in the range of 6.5 to 6.6, depending upon how the titration end-point is chosen, close to the experimental value of 6.42 for MPEG-S-BR [9].

Our models for UCB, which include aggregates, thus provide some qualitative rationalization for the very unusual features of the titration curves for MPEG-S-BR [9]. The comparison is dependent upon the assumptions that: a) the bilirubin moiety in MPEG-S-BR behaves essentially like UCB [9]; b) the MPEG-S-BR used was reasonably pure; and c) the low equivalence value determined in the NaOH titration of MPEG-S-BR is due to premature identification of the end point. We emphasize that our models are based on the current limited understanding of the self-association of UCB, and can only be qualitative. It is clear, however, that, since all our models use pKa values of 8.1 and 8.4 for monomeric UCB, self-association leads to a reduction in the apparent average pKa values that are estimated from the pH at the mid-point of the titration. If the self-association behavior of MPEG-S-BR is similar to that of UCB, the reported average pKa of 6.4 for MPEG-S-BR, derived from the titration midpoint [9], must be **lower **than the true pKa values of monomeric MPEG-S-BR and is thus qualitatively inconsistent with low pKa values for UCB. If MPEG-S-BR is not similar to UCB, no conclusions can be drawn regarding UCB. The extraordinary aspects of the modeled titration curves may also be of some usefulness in the understanding of the acid titrations of high concentrations of sodium salts of UCB [47].

In summary, we have pointed out the many experimental problems associated with the acid-base titration of MPEG-S-BR and MPEG-FAs [9], and serious dissimilarities of the titration curves of MPEG-FAs and MPEG-S-BR. The former can be explained by classical electrostatic theories and are inappropriate models for the latter. In view of the very high concentrations of MPEG-S-BR used for titration, we have modeled the effects of self-aggregation of UCB on its titration curves and the apparent pKa of about 6.4, derived from the mid-point of the curve [9] (see appendix). Our modeling thus suggests that the pKa values of monomeric MPEG-S-BR may be similar to the high values of 8.1 and 8.4 for UCB. The data for MPEG-S-BR clearly provide no acceptable evidence for low pKa values of monomeric UCB itself.

## Conclusions

### Summary

We have summarized estimates of bilirubin pKa values derived from published potentiometric or spectroscopic titrations, dissolution of UCB crystals, HNP measurements in DMF, co-titrations in DMSO coupled with the use of the Born equation, and the recent estimates from NMR data of [^13^C]-mesobilirubin-XIIIα in water-(CD_3_)_2_SO mixtures and the titration of MPEG-S-BR in water (Additional file 1, Table S1). We have shown all those estimates to be unreliable, due to failure to fulfill one or more of the minimum criteria for a valid study, as well as other serious deficiencies that confound interpretation of those studies. As summarized in Additional file 1, Table S1, only our solvent partition studies [10,11] have met all the requirements for valid experiments when using a poorly-soluble, unstable compound, such as UCB, and these studies clearly indicate that the pKa values of UCB are well above the mean pKa values of simple mono- and di-carboxylic acids (below 5.0) [28,31]. We also note that a critical evaluation of the theoretical basis used in deriving aqueous pKa values in water from experiments in DMF [26] and DMSO [27,29,37] reveals serious deficiencies. The new analysis gives rise to new pKa estimates: the average pKa of UCB in DMSO is about 11.1 and in DMF it is above 10. These values are consistent with the high pKa values of UCB in water [10,11]. Some models used to rationalize unusual titration behavior of MPEG-S-BR indicate an important general concept: reversible self-association of UCB-type molecules, involving primarily dianions, can lead to falsely low estimates of pKa values and can generate some unusual titration curves.

### Concluding remarks

We first highlight the major factors involved in the reporting of low, intermediate and high pKa values for UCB. We have detailed many different reasons for deciding why most of the pKa values in Additional file 1, Table S1 are unreliable. A general point, applicable especially to the spectral studies in water (Additional file 1, Table S1), is that acidification of true solutions of UCB, initially dissolved at a high pH, can cause massive supersaturation, followed by formation of colloidal and coarser species, which themselves cause spectral changes [33-35]. The net effects of these non-equilibrium processes may depend upon time, concentration, impurities and, most importantly, on how low the pH becomes. These variable factors, particularly the effects of pH on the formation of aggregates, largely explain the serious discrepancies among the various reported pKa values (Additional file 1, Table S1), as well as their relatively low magnitude (clearly illustrated in Figure 1).

Potentiometric titration has shown that most of the neutralization by added acids, of UCB dissolved at a high pH, occurs between pH 8 and 7 [35]. Leaving aside the two lowest of the extraordinary set of four pKa values reported by Kolosov and Shapolovenko [21], the average experimental pKa values from four spectrophotometric studies [20-23] and one potentiometric titration [24] (Additional file 1, Table S1) lie between 6.8 and 7.6. The average pKa values of 6.8 [22] and 8.1 [25], derived from dissolution of crystals of UCB diacid, are moderately high, but unacceptable for reasons described above and by Hahm, *et al. *[10]. The very low average pKa values of about 4.6, promulgated frequently in recent years, have been derived, directly or indirectly, mostly from studies in non-aqueous media [26-31], or using a water-soluble, thioether conjugate of UCB [9]. Their deficiencies have been detailed above and summarized in Additional file 1, Table S1. The high aqueous pKa values of 8.12 ± 0.23 and 8.44 ± 0.33 (mean ± S.D.), that we have concluded to be reliable, derive from partition of UCB between aqueous solutions and chloroform [10,11]. Uniquely, these two studies were designed to avoid problems of supersaturation, precipitation and degradation of UCB during prolonged procedures.

### Rationale for and significance of high pKa values of UCB

In our earlier papers [1,10,25], the high pKa values of UCB were attributed to hydrogen-bonding interactions, without detailed rationalization. Our recent study, with [^14^C]-UCB [11], confirmed our original solvent partition data using unlabeled UCB [10], and postulated three factors that, collectively, could explain the remarkably high pKa values of 8.12 and 8.44 derived from these experiments. Each factor was related to the crowded and constrained microenvironment created for each -COOH or ionized -COO^- ^group in UCB, by the unique, multiple intramolecular H-bonds involving these groups [62]. These factors are [11]: a) donation of an H-bond from the -OH moiety of the -COOH group; b) hindered solvation of the -COO^- ^group; and c) restricted rotation of the -COO^- ^and -COOH groups, which also contributes to suboptimal solvation. The evidence for these effects on the suppressed ionization of the -COOH groups in UCB has been detailed elsewhere [11].

These theoretical rationalizations indicate that such remarkably high pKa values are not unreasonable [11], and dictate that the freely diffusible UCB diacid (H_2_B) [1], rather than the dianion (B^=^) [63], is the predominant unbound species of UCB in plasma at physiological pH. The implications for understanding UCB cytotoxicity and bilirubin encephalopathy in jaundiced neonates have been discussed elsewhere [3,64].

## Abbreviations

UCB: unconjugated bilirubin; B_T_: total UCB concentration; H-bond: hydrogen-bond; H_2_B: UCB diacid; HB^-^: UCB monoanions; B^=^: UCB dianion; R: the UCB saturation ratio = free UCB concentration/estimated solubility of UCB at a given pH; DMF: dimethyl formamide; HNP: half-neutralization potential; NMR: nuclear magnetic resonance; MPEG: polyethylene glycol monomethyl ether; MPEG-S-BR: thioether conjugate of MPEG with UCB; MPEG-FA: monoester conjugate of MPEG with a dicarboxylic fatty acid; (CD_3_)_2_SO: deuterated dimethylsulfoxide; CDCl_3_: deuterated chloroform; PEG: polyethylene glycol; c.m.c.: critical micellar concentration.

## Authors' contributions

Both authors were equally involved in the conceptualization and writing of this paper, and both have read and approved the initial and revised manuscript. JDO performed the literature search and PM developed the mathematical models.

## Appendix

### Simulation of the Effects of Self-Association on Titration Curves of UCB

The titration curves in Figure 1 are represented by plots of pH vs. F, where F is the ratio of equivalents of NaOH added to the total equivalents of UCB or MPEG-S-BR. The total concentration of UCB or MPEG-S-BR in solution is given by Equation 5.(5)

The particular model of aggregation chosen for simulation of the titration curve determines which of the above aggregates are selected for inclusion in Equation 5 (see below). The corresponding aggregate species must also be selected for Equation 6, which represents the concentration of Na^+ ^in the system.(6)

In Equations 5 and 6, all the terms on the right hand side can be represented by the equilibrium concentrations of [B^=^] and [H^+^]. The chosen values of pK1 = 8.1 and pK2 = 8.4 [10] and the pKw value of 10^-14^, can be used to calculate [H_2_B], [HB^-^] and [OH^-^]. The models assume that the role of the MPEG moiety is negligible, so that our previously estimated constants for UCB [10] also apply to MPEG-S-BR. The equilibria described below for the formation of aggregates allow their concentrations to be determined from the equilibrium values of [HB^-^] + [B^=^] and, therefore, from [B^=^] and [H^+^], using the equilibrium constants chosen for the simulations. Different known values of BT and [Na^+^] were generated in the progressive titration of 25 mL of 0.007 M UCB with 1.06 × 10^2 ^M NaOH, up to and beyond neutralization, taking volume changes into account. The concentrations chosen are similar to those used for titration of MPEG-S-BR with NaOH by Boiadjiev *et al. *[9]. Equations 5 and 6 were solved for the two unknowns, the equilibrium values [B^=^] and [H^+^], using the SCIENTIST program (Micromath Scientific Software, Salt Lake City, UT). The equilibrium pH values so determined have been plotted against F in Figures 1 A & B, neutralization being represented by F = 1.

Four different models (A-D), with increasingly complex self-association patterns, have been examined to determine how some of the characteristic features of the titration are affected by self-association of [B^=^]. All concentrations were in mol/L units, and the assumed equilibrium constants had, therefore, units consistent with this.

Model A - No aggregation of B^= ^in Equations 1 and 2. BT = [H_2_B] + [HB^-^] + [B^=^].

Model B - The aggregation is limited to the dianion dimer, (B^=^)_2_, so that

BT = [H_2_B] + [HB^-^] + [B^=^] + 2[(B^=^)_2_]. The dimerization constant, KD, for the equilibrium 2B^= ^↔ (B^=^)_2_, has been estimated as 2.6 × 10^5 ^M^-1 ^[10] and 6.7 × 10^5 ^M^-1 ^[47]. We have chosen, conservatively, the lower value (log KD = 5.415, rather than 5.826).

Model C - We assume the formation of (B^=^)_2 _and the pentameter, (B^=^)_5_. Therefore, BT = [H_2_B] + [HB^-^] + [B^=^] + 2[(B^=^)_2_] + 5[(B^=^)_5_]. In the absence of any co-operativity, the value of K5, the equilibrium constant governing pentamer formation, 5B^= ^↔ (B^=^)_5_, should be (KD)^4^. Since some co-operativity is expected, we have used log K5 = 4 log KD + 1 = 22.66, assuming KD has the lower value of 2.6 × 10^5 ^M^-1 ^[10].

Model D - For larger aggregates, the formation of heteromers is likely [47]. In this model, we have assumed the presence of three mixed adducts of HB^- ^and B^= ^containing 8 monomers, (H^+^)_3_(B^=^)_8_, (H^+^)_4_(B^=^)_8_, and (H^+^)_5_(B^=^)_8_, along with the dimer, (B^=^)_2_. B_T_ = [H_2_B] + [HB^-^] + [B^=^] + 2[(B^=^)_2_] + 8[(H^+^)_3_(B^=^)_8_] + 8[(H^+^)_4_(B^=^)_8_] + 8[(H^+^)_5_(B^=^)_8_]

Significant co-operativity is expected in the formation of these larger species, but the self-association of HB^- ^is expected to be less favorable than the self-association of B^= ^[10,47]. If there is no co-operativity of self-association, and if HB^- ^and B^= ^exhibit the same tendency to self-associate, the K value controlling the formation of the octameric species from the monomeric species should be given by (KD)^7^, so that log K = 37.9 for the lower value of KD (2.6 × 10^5 ^M^-1^) [10] and log K = 40.8 for the higher value of KD (6.7 × 10^5 ^M^-1^) [47]. Co-operativity effects should increase log K by a few units. To accommodate both effects, we have selected log K(H^+^)_3_(B^=^)_8 _= 40 for the equilibrium 3 HB^- ^+ 5 B^= ^↔ (H^+^)_3_(B^=^)_8_, log K(H^+^)_4_(B^=^)_8 _= 39 for 4 HB^- ^+ 4 B^= ^↔ (H^+^)_4_(B^=^)_8_, and log K(H^+^)_5_(B^=^)_8 _= 38 for 5 HB^- ^+ 3 B^= ^↔ (H^+^)_5_(B^=^)_8_. The log K values represent some contribution of co-operativity. The progressively lower K values in the sequence log K(H^+^)_3_(B^=^)_8 _> log K(H^+^)_4_(B^=^)_8 _> log K(H^+^)_5_(B^=^)_8 _represent the expected weaker association of HB^- ^compared with B^=^. The increasing net charge of the aggregates which contain fewer HB^- ^will tend to mitigate this effect somewhat.

The key results of these simulated titrations are given in the text and Figure 1.

## Supplementary Material

Additional file 1 **Derived pKa values of bilirubin in simple systems**. Details of the 18 studies from 17 publications that were considered, including the degrees of supersaturation with UCB, the analytical methods used, the apparent pKa values, the experimental problems, and the citation. Citation numbers correspond to those in the list of references in the manuscript. Click here for file
