# Supplementary material for: Review: Bilirubin pKa studies; new models and theories indicate high pKa values in water, dimethylformamide and DMSO
Source: BMC Biochem. 2010 Mar 29;11:15. doi: 10.1186/1471-2091-11-15 (PMC2880415; doi:10.1186/1471-2091-11-15)
Supplement: Additional file 1 — Derived pKa values of bilirubin in simple systems. Details of the 18 studies from 17 publications that were considered, including the degrees of supersaturation with UCB, the analytical methods used, the apparent pKa values, the experimental problems, and the citation. Citation numbers correspond to those in the list of references in the manuscript. [file 1471-2091-11-15-S1.DOC]

**Table S1. Derived pK*a*** values of bilirubin in simple systems

| **Solvent** | **Aq. saturation ratios (R)**  **for bilirubin**^1^ | **Method** | **Apparent**  **pK*a* values**^2^ | **Experimental**  **Problems**^3^ | **Citation** |
| --- | --- | --- | --- | --- | --- |
| H_2_O + MeOH (8:1 v/v) | R~73 at pH 7.0, ~38 at pH 8.0 | Visible Spectra vs. pH | 7.1*^a,b^* | A,B,C,D | Gray *et al*., 1961 [20] |
| H_2_O | R>172 at pH 7.0, >91 at pH 8.0 | Visible Spectra vs. pH | pKa_1_=4.5, pKa_2_=5.9,  Mean pKa_3&4_ =7.3*^c^* | A,B,C,D | Kolosov & Shapolovenko, 1977 [21] |
| H_2_O | R~25 at pH 7.0, ~13 at pH 8.0 | Visible Spectra vs. pH | 6.2 & 7.4 | B,C | Moroi *et al*., 1985 [22] |
| H_2_O | R=109 at pH 7.0, 58 at pH 8.0 | Visible & Raman Spectra vs. pH | 6.0 & 8.3 | A,C,D | Russell *et al*., 1995 [23] |
| H_2_O | R = 4886 at pH 7.0 | Potentiometric titration with both NaOH and HCl | 4.4 & 5.0*^d,e^* | C,E | Overbeek *et al*., 1955 [16] |
| H_2_O +  Triton X-100 | R = 2.4–8.1 x10^4^ at pH 7.0 Precipitation at pH < 8 | Potentiometric titration with both NaOH and HCl | 7.55*^a,b,e^* | A,C,F | Krasner & Jaffe, 1973 [24] |
| H_2_O | MPEG-S-BR studied. Undersaturated, but marked self-aggregation | Potentiometric titration of MPEG-S-BR | 6.4 | G,H,I | Boiadjiev *et al*., 2004 [9] |
| H_2_O | Marked supersaturation | Crystal solubility vs. pH | 6.0 & 7.6 | B,C,F | Moroi *et al*., 1985 [22] |
| H_2_O | Marked supersaturation | Crystal solubility vs. pH | 6.8 & 9.3 | C,F | Ostrow *et al*., 1988 [25] |

(Table S1 continued on next page)

| **Solvent** | **Aq. saturation ratios (R)**  **for bilirubin**^1^ | **Method** | **Apparent**  **pK*a* values**^2^ | **Experimental**  **Problems**^3^ | **Citation** |
| --- | --- | --- | --- | --- | --- |
| (CH_3_)_2_NCHO | Probably undersaturated | Potentiometric titration, with half-neutralization potentials | 4.3 & 5.3 | A,G | Lee *at al*., 1974 [26] |
| (CD_3_)_2_SO | Always undersaturated | [^13^C]-NMR, titration | 4.4 *^a,,f^* | A,B,G,J | Hansen *et al*., 1979 [27] |
| H_2_O-(CD_3_)_2_SO | Many systems supersaturated (see Mukerjee & Ostrow [12]) | NMR changes in [^13^C]OOH groups vs. pH | 4.2 & 4.9 | B,C,G,K,L | Lightner’s laboratory, 1996-1999 [28-31] |
| Buffered H_2_O | Aq. phase supersaturated at all pH. R > 50 at pH 7.6. | Partition into n-heptane + methyl iso-butyl ketone. Data excluded if visible precipitates present. | 4.4 & 5.0*^d^* | A,B,C,E,M | Irollo, 1979 [32] |
| Buffered H_2_O | Always undersaturated | Partition from CHCl_3_ vs. pH. Diazo assay. | 8.1 & 8.4 |  | Hahm *et al*., 1992 [10] |
| Buffered H_2_O | Always undersaturated | Partition from CHCl_3_ vs. pH. Radioassay. | [^14^C]-UCB partitions confirmed Hahm's results [10] |  | Ostrow & Mukerjee, 2007 [11] |

**Abbreviations:** UCB, unconjugated bilirubin; (CH_3_)_2_NCHO, dimethylformamide; (CD_3_)_2_SO, deuterated DMSO; FTIR, Fourier transform infrared spectroscopy; MPEG-S-BR, thioether conjugate of polyethylene glycol monomethyl ether with UCB.

**Footnotes:**

1 R calculated as ratio to saturation concentration for UCB at given pH, based on data from Hahm *et al.,* 1992 [10]

2 Apparent pK*a* values assessed from midpoint of data curves if authors did not determine one.

3 *Experimental Problems* as listed below:

A- UCB not purified and/or its purity not documented

B- UCB not protected against degradation by exclusion of oxygen and light, removal of oxidants from solvents, and avoidance of alkaline stock solutions.

C- UCB concentrations greatly above saturation, with aggregation or precipitation, and/or soluble multimers not accounted for.

D- Sketchy experimental details. I– Titration equivalence endpoints misidentified.

E- Assume low pKa’s in analyzing data J- Incorrect pKa values used for reference carboxylic acids.

F- Equilibrium not achieved and/or not attained rapidly. K- Used prolonged analytical procedures.

G- No direct measurements of UCB pKa in aqueous systems. L- pH measurements not standardized in mixed solvents.

H– Marked self-aggregation of MPEG-S-BR. M- Solvents not purified.

a- Mean values for pKa_1_ & pKa_2_.

b - This has been interpreted by some as the sum of pKa_1_ & pKa_2_.

c - The highest pKa's were associated with the dominant spectral changes.

d - These pK'a values were not derived from the data, but were assumed by the investigators and then modeled to the data.

e - Similar data has been reported by Lucassen [38] and Carey & Spivak [35], who likewise found extensive precipitation below pH 8.0 to 8.3, with most of the neutralization occurring between pH 8.0 and 7.0. Those authors felt that pKa values could not be reliably derived from such data obtained in the presence of a variable phase of precipitated bilirubin.

f - Calculated, using the Born equation, from comparison of titration curves of bilirubin and *m*-hydroxybenzoic acid in DMSO.
